# Alternative initiations with 6-monthly paliperidone palmitate. A retrospective study

**DOI:** 10.1192/j.eurpsy.2023.1044

**Published:** 2023-07-19

**Authors:** S. Benavente López, S. Bolaño Mendoza, A. Parra González, A. Lara Fernández, A. Herencias Nevado, E. Baca García

**Affiliations:** Psychiatry, Hospital Universitario Infanta Elena, Madrid, Spain

## Abstract

**Introduction:**

6-monthly paliperidone palmitate features an initiation regimen through 1-monthly paliperidone palmitate or 3-monthly paliperidone palmitate. Some patients do not have sufficient adherence to treatment and it is necessary at the clinical level to start directly with 6-monthly paliperidone palmitate. There is little clinical experience with these alternative initiations and through this work those that have been carried out for 12 months at the Hospital Universitario Infanta Elena are exposed.

**Objectives:**

The main objective of the study is to describe the alternative initiations performed with 6-monthly paliperidone palmitate in routine clinical practice, having opted for a regimen different from the standard for clinical reasons.

**Methods:**

A retrospective selection of patients will be made through non-probabilistic consecutive sampling, including all patients who have been administered 6-monthly paliperidone palmitate with a start different from the standard during the last 4 months. To do this, the electronic medical record will be used, first selecting the patients who have started 6-monthly paliperidone palmitate through the anonymized digital records and, later, including in the study only those who have followed an alternative initiation pattern. The variables studied will be the following: age, sex, diagnosis, dose of paliperidone palmitate, initiation regimen, consumption of toxic substances, absenteeism from 6-monthly paliperidone palmitate, and visits to the emergency room and admissions.

**Results:**

The study included a total of 20 patients (n: 20). 80% of the patients were male and 20% were female. The mean age was 39.7 years. 75% of the patients had an associated substance use disorder. The following alternate starting schedules were performed with biannual paliperidone palmitate: monthly paliperidone palmitate on days 1 and 8, and 6-monthly paliperidone palmitate on day 38 (n: 11); monthly paliperidone palmitate 150 mg together with semi-annual paliperidone palmitate both on day 1 (n: 5); biannual paliperidone palmitate on day 1 supplemented with oral paliperidone for 45 days (n:4). A total of 0 visits to the emergency department and 0 admissions were observed after the 6-monthly paliperidone palmitate regimen.

**Conclusions:**

Alternative initiations with 6-monthly paliperidone palmitate may be a useful and safe clinical alternative in patients with very low adherence who, due to clinical needs, require starting 6-monthly paliperidone palmitate earlier in order to guarantee adherence.

**Disclosure of Interest:**

None Declared

